# Later Response to Corticosteroids in Adults With Primary Focal Segmental Glomerular Sclerosis Is Associated With Favorable Outcomes

**DOI:** 10.1016/j.ekir.2021.10.016

**Published:** 2021-10-29

**Authors:** Ilse M. Rood, Aernoud Bavinck, Beata S. Lipska-Ziętkiewicz, Dorien Lugtenberg, Franz Schaefer, Jeroen K.J. Deegens, Jack F.M. Wetzels

**Affiliations:** 1 Department of Nephrology, Radboud University Medical Center, Radboud Institute for Health Sciences, Nijmegen, The Netherlands; 2 Rare Diseases Centre and Clinical Genetics Unit, Department of Biology and Medical Genetics, Medical University of Gdansk, Gdansk, Poland; 3 Department of Human Genetics, Radboud University Medical Center, Radboud Institute for Health Sciences, Nijmegen, The Netherlands; 4 Division of Pediatric Nephrology, Center for Pediatrics and Adolescent Medicine, Heidelberg, Germany

**Keywords:** corticosteroids, focal segmental glomerulosclerosis, nephrotic syndrome, steroid resistant nephrotic syndrome

## Abstract

**Introduction:**

Guidelines advise initial therapy with corticosteroids (CSs) in patients with presumed primary focal segmental glomerular sclerosis (pFSGS). Many patients do not achieve complete remission (CR) after 8 or 16 weeks. Although these patients are considered steroid resistant, clinical outcomes are ill defined.

**Methods:**

A retrospective cohort study of patients with pFSGS who were referred between January 1995 and December 2014. Data of clinical presentation until last follow-up were collected from patient records.

**Results:**

A total of 51 patients (median age 47 years, 20 female/31 male) were included (median follow-up 7.1 years). There were 10 patients who achieved partial response (PR) at 8 weeks. High-dose CS monotherapy was continued for a median of 17 weeks (interquartile range [IQR] 11–21 weeks) (total duration 56 weeks [IQR 28–83 weeks]). With CSs, the cumulative incidence of CR + PR was 18% and 35%, respectively. Of 24 patients with persistent nephrotic-range proteinuria, 22 received additional immunosuppressive (IS) therapy, resulting in CR in 3 (14%) and PR in 11 patients (50%). A decrease of >20% of proteinuria at 8 weeks predicted response. In addition, 8 patients (36%) were considered primary nonresponders. A genetic cause was found in 2 patients. Proteinuria at end of follow-up was 1.2 g (IQR 0.4–3.0 g/24 hours or g/10 mmol creatinine). Renal survival at 3, 5, and 10 years was 92%, 87%, and 64%, respectively.

**Conclusion:**

Patients with presumed pFSGS often respond late to IS therapy. A decrease in proteinuria of >20% after 8 weeks of therapy is a predictor of responsiveness. Regardless of CR in some patients, improved biomarkers are needed to predict response/outcomes in patients with pFSGS.

## Graphical abstract

**Figure:**
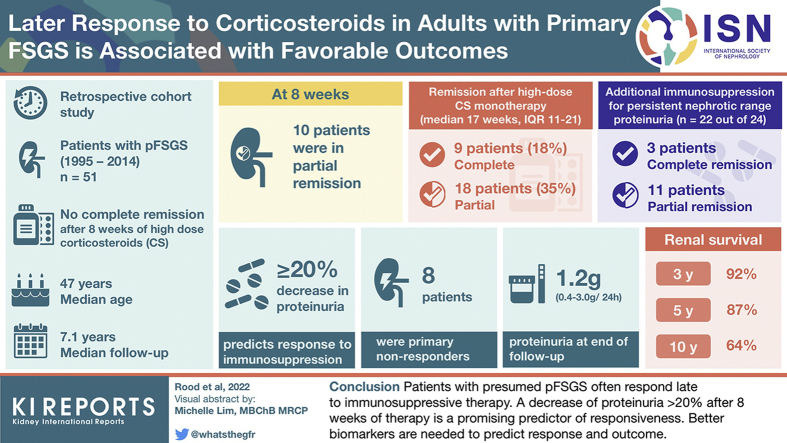

See Commentary on Page 9

FSGS is one of the most common patterns of glomerular injury in adult patients with nephrotic syndrome (NS). It is not a disease but merely a description of glomerular abnormalities observed in the kidney biopsy findings. Etiologic classification is important for therapy, prognosis, and counseling.^1^^,^^2^ FSGS can be secondary to many underlying causes such as genetic defects (genetic FSGS), infections, and medication or maladaptive responses owing to hypertension or obesity. pFSGS remains a clinical (“presumed”) diagnosis, and it is defined by the presence of NS with low serum albumin levels and complete foot process effacement, in the absence of underlying secondary causes (such as small kidney size, reflux nephropathy, and a positive family history of obesity). pFSGS is attributed to a circulatory permeability factor, although the causative factor has not been identified. The clinical course is variable: few patients develop spontaneous remission,^3^ whereas others progress to have end-stage renal disease. To induce remission and to avoid kidney function deterioration, patients with pFSGS are treated with high-dose CSs. Nevertheless, response to treatment with CSs is unpredictable. Only 21% to 40% of the treated patients attain a CR.^4^, ^5^, ^6^, ^7^ Patients who are steroid unresponsive are considered steroid resistant, but there is no consensus in literature regarding the duration of prednisone therapy that defines steroid resistance.^8^ In children, steroid resistance is defined as no response within 4 to 8 weeks of treatment with high-dose CSs.^9^ In adults, some authors define steroid resistance as persistent NS after 8 weeks of treatment with high-dose CSs.^10^ Others describe remissions in patients after prolonged treatment with high-dose CSs and argue that steroid resistance should be defined after a treatment duration with high-dose CSs of up to 4 months.^8^^,^^11^, ^12^, ^13^, ^14^ The variation in definitions used to define steroid resistance hampers the interpretation of clinical course and outcome. Moreover, several studies included patients without overt NS (i.e., without hypoalbuminemia).^4^^,^^5^^,^^15^ These patients do not fulfill the definition of presumed pFSGS, and the recently published guideline of the Kidney Disease: Improving Global Outcomes now includes the category FSGS of undetermined cause.^2^^,^^16^ It is generally accepted that patients with secondary forms of FSGS are unresponsive to CSs.

In this study, we retrospectively analyzed a well-characterized cohort of patients with NS (defined as proteinuria ≥ 3.5 g/24 hours or ≥3.5 g/10 mmol creatinine and serum albumin ≤ 30 g/l) owing to presumed pFSGS, who did not attain a CR after 8 weeks of treatment with high-dose CSs. We investigated the following: (i) response to continued treatment with CSs; (ii) histologic and clinical markers that are associated with response to treatment/outcome; (iii) response to second-line treatment; and (iv) long-term outcome.

## Methods

### Study Design and Patient Selection

A retrospective study was performed in patients with presumed pFSGS. From the pathology registry, we identified adult patients with a diagnosis of FSGS who were referred to the Radboudumc (a tertiary hospital) between January 1995 and December 2014 (FSGS cohort). Histologic criteria for FSGS were glomerular abnormalities according to the Columbia classification as previously described.^17^ Data of clinical presentation until last follow-up were collected from patient records.

All patients with NS (at kidney biopsy and/or start of treatment) who were initially treated with high-dose CSs (1 mg/kg/d or 2 mg/kg every other day) were included in the current study. Exclusion criteria were onset of NS <16 years, attaining CR of NS within 8 weeks of treatment, missing treatment data, follow-up period <6 months (unless the patient died or developed end-stage renal disease [ESRD] within 6 months), or a known/suspected secondary cause of FSGS using clinical criteria, such as a history of kidney injury, obesity, or a positive family history for kidney disease.

Patients were treated by their nephrologist. In the study period, high-dose CSs was the standard of therapy. The duration of high-dose CSs and the decision to start second-line IS therapy were not protocolized. Nevertheless, in the study period, most centers were in favor of giving long-term IS therapy. Total dosage of CSs was estimated based on date of start of CSs, date of tapering, date of end of therapy, and the prescribed dosing schedules.

According to local and national policies, ethics approval is not required for retrospective research with patient files. Chart review was performed under the supervision of physicians who were involved in the treatment of patients. All data were anonymized. The study was conducted according to the Code of Conduct for Medical Research.

### Definitions

NS was defined as proteinuria level ≥3.5 g/24 hours or ≥3.5 g/10 mmol creatinine and serum albumin ≤30 g/l. (In 22 patients, serum albumin level was measured using bromocresol green, in 20 patients bromocresol purple, and in 9 patients, the type of test was unknown.) CR was defined as proteinuria level ≤0.3 g/24 h or ≤0.3 g/10 mmol creatinine with progressively increasing serum albumin levels. PR was defined as proteinuria level of 0.3 to 3.5 g/24 hours or 0.3 to 3.5 g/10 mmol creatinine and a decrease in proteinuria level of at least 50% from baseline with stable serum creatinine level (<25% increase from start of therapy). Primary nonresponse was defined as absence of remission (PR/CR) after high-dose CSs and second-line IS therapy.

Time to remission was measured from start of therapy to the first day that remission was observed. Relapse was defined as proteinuria level ≥3.5 g/24 hours or ≥3.5 g/10 mmol creatinine after PR or CR was reached. Indication for a second course of IS treatment was classified as either presumed sustained active disease (no CR) or recurrence of NS (relapse). ESRD was defined as need for nontemporary renal replacement therapy, persistent serum creatinine concentration ≥450 μmol/l, or CKD stage G5 (estimated glomerular filtration rate [eGFR] ≤15 ml/min per 1.73 m^2^). eGFR was calculated using the CKD-epi formula. Acute kidney injury was defined according to the Risk, Injury, Failure, Loss of kidney function, and End-stage kidney disease criteria.^18^ Creatinine levels before onset of NS were not available in many patients. If not available, to generate baseline levels to define acute kidney injury, the eGFR at presentation was compared with median age- and sex-based reference values.^19^ Progression of kidney dysfunction was defined as occurrence of ESRD or increase in at least 1 CKD stage and >15 ml/min per 1.73 m^2^ deterioration of kidney function. Hypertension was defined as either systolic blood pressure ≥140 mm Hg, diastolic blood pressure ≥90 mm Hg, or the use of antihypertensive medication.

### Genetic Analysis

#### Routine Diagnostics

Sanger sequencing of *NPHS2* was performed in 2 patients in daily clinical practice.^20^ DNA was extracted from whole blood samples and enriched with the Agilent SureSelectXT Human All Exon 50Mb Kit (Santa Clara, CA). Amplicons harboring the variants of interest were amplified using exon-specific polymerase chain reaction primers. Polymerase chain reaction was performed using AmpliTaq Gold DNA polymerase on a GeneAmp polymerase chain reaction 9700 system. After purification using Millipore plates, Sanger sequencing was performed using an ABI3730XL platform (Thermo Fisher Scientific, Waltham, MA).

In 4 patients, exome sequencing was performed. DNA was extracted and enriched as described previously. Sequencing was performed using an Illumina HiSeq2000 or HiSeq4000 machine (San Diego, CA) at BGI-Europe in Copenhagen, Denmark. After read alignment with BWA and variant calling with GATK, variants were annotated using software developed at the Department of Genetics of the Radboudumc. In these 4 patients, only genes in the kidney disorder gene panel (up to 250 genes at time of genetic testing) were evaluated. Data on frequency of variants in control populations (<5% in dbSNP and <1% in an in-house database [with an exception for the relatively frequent non-neutral p.R229Q polymorphism in *NPHS2*]), nucleotide and amino acid conservation, inheritance pattern, and the phenotype associated with the genes were combined to prioritize variants.

#### Research

As part of a separate study concerning genetic FSGS, patients of the current cohort with available DNA (*n* = 18) were tested for genetic variants using a custom-designed multiplex polymerase chain reaction kit (MASTR FSGS, Multiplicom, Niel, Belgium) as described before.^21^^,^^22^ DNA was extracted as described previously. By targeted enriched sequencing, 37 known or plausible steroid-resistant NS/FSGS genes including also collagen IV (Alport) genes were analyzed.

Informed consent for analysis of the DNA samples was obtained from all 18 patients. This study was approved by the Radboudumc Medical Review Ethics Committee (2015-2108).

One patient was tested for mitochondrial mutations. This case (including methods of testing) has been published before.^23^

### Statistics

Continuous variables are expressed as mean ± SD in case of a normal distribution of the data and otherwise as median and IQR. Categorical variables are expressed as a number and percentage. The independent sample *t* test and Mann-Whitney *U* test were used to compare continuous variables as appropriate, and the χ^2^ test was used for categorical variables. Time to remission was analyzed using the Kaplan-Meier method, and *P* values were generated with the log-rank test. A *P* < 0.05 was considered statistically significant. Statistical analysis was performed with SPSS version 22.

### Review of Literature

For comparison of baseline characteristics and outcomes of the current study, we performed a systematic search in the PubMed database for studies (and their references) with adult patients with FSGS who were treated with CSs published until January 2021. The following search terms were used: (“Glomerulosclerosis, Focal Segmental” [Mesh] OR “focal segmental glomerulosclerosis” [tiab] OR “focal and segmental glomerulosclerosis” [tiab] OR “FSGS” [tiab]) AND (“Nephrotic Syndrome” [Mesh] OR “nephrotic syndrome” [tiab]) AND (“Glucocorticoids” [Mesh] OR “glucocorticosteroids” [tiab] OR “corticosteroids” [tiab] OR “prednisone” [tiab]) AND (“Adult” [Mesh] OR “adult”). Studies describing treatment with CSs, response to treatment, and/or outcome in patients with suspected pFSGS were included. Case reports, reviews, and studies that did not describe course of treatment with CSs were excluded. There were 132 publications that were identified by the initial search and evaluated by title and abstract. In total, 11 studies were selected.

## Results

### Patients

From the FSGS cohort (*n* = 190), we identified 70 patients who presented with NS and were initially treated with high-dose CSs as monotherapy. There were 2 patients excluded (1 patient with a functional monokidney, 1 patient had a multiple myeloma with depositions in kidney biopsy findings). In addition, 15 patients were excluded because of insufficient follow-up. Baseline characteristics of these excluded patients did not differ from those of the included patients (data not shown). We excluded 2 patients who attained a CR of NS within 8 weeks of treatment. In total, 51 patients (all White) with persistent proteinuria at 8 weeks were included in the current study. No underlying cause was identified, and family history for kidney diseases was negative. Kidney biopsy results revealed FSGS subtype not otherwise specified in 25 patients (49%), FSGS-tip in 20 patients (40%), FSGS-collapsing in 2 patients, FSGS-cellular in 1 patient, and unknown subtype in 3 patients. Of note, in no patient the perihilar variant was observed. Complete foot process effacement was present in 27 of 29 kidney biopsies that were evaluated by electron microscopy. Baseline characteristics at time of kidney biopsy are found in Table 1.

**Table 1:** Patient characteristics at time of kidney biopsy

Characteristics	Cohort
Total (*n*)	51
Age (yr)	46.7 (31.8–58.3)
Gender (male/female)	31/20
BMI (kg/m^2^)	26.0 (24.3–29.5)
Hypertension	47 (94)
AKI	11 (22)
Serum creatinine (μmol/l)	94.0 (77.3–141.0)
Serum creatinine no AKI	89.0 (73.0–102.5)
Serum creatinine AKI	175.5 (106.3–212.3)
eGFR^a^ (ml/min per 1.73 m^2^)	77 (52–98)
eGFR no AKI	85 (62–101)
eGFR AKI	38 (25–54)
Serum albumin (g/l)	20.9 ± 6.1
Urinary protein excretion (g/24 h or g/10 mmol creatinine)	8.7 (6.3–12.7)
Histologic features	
Interval between onset of symptoms and biopsy (d)	112 (34–144)
Complete podocyte foot process effacement at EM^b^	27 (93)
Pathologic FSGS variant	
NOS	25 (49)
Tip	20 (39)
Collapsing	2 (4)
Cellular	1 (2)
Perihillar	0 (0)
Not defined	3 (6)

AKI, acute kidney injury; BMI, body mass index; CKD, chronic kidney disease; CKD-EPI, Chronic Kidney Disease Epidemiology Collaboration; eGFR, estimated glomerular filtration rate; EM, electron microscopy; FSGS, focal segmental glomerular sclerosis; IQR, interquartile range; NOS, not otherwise specified.

Values are presented as *n* (%), mean ± SD or median (IQR). AKI was defined by RIFLE criteria.

a eGFR was estimated with the CKD-EPI equation.

b EM available in 29 patients.

### Initial Treatment and Outcome

**Figure 1:**
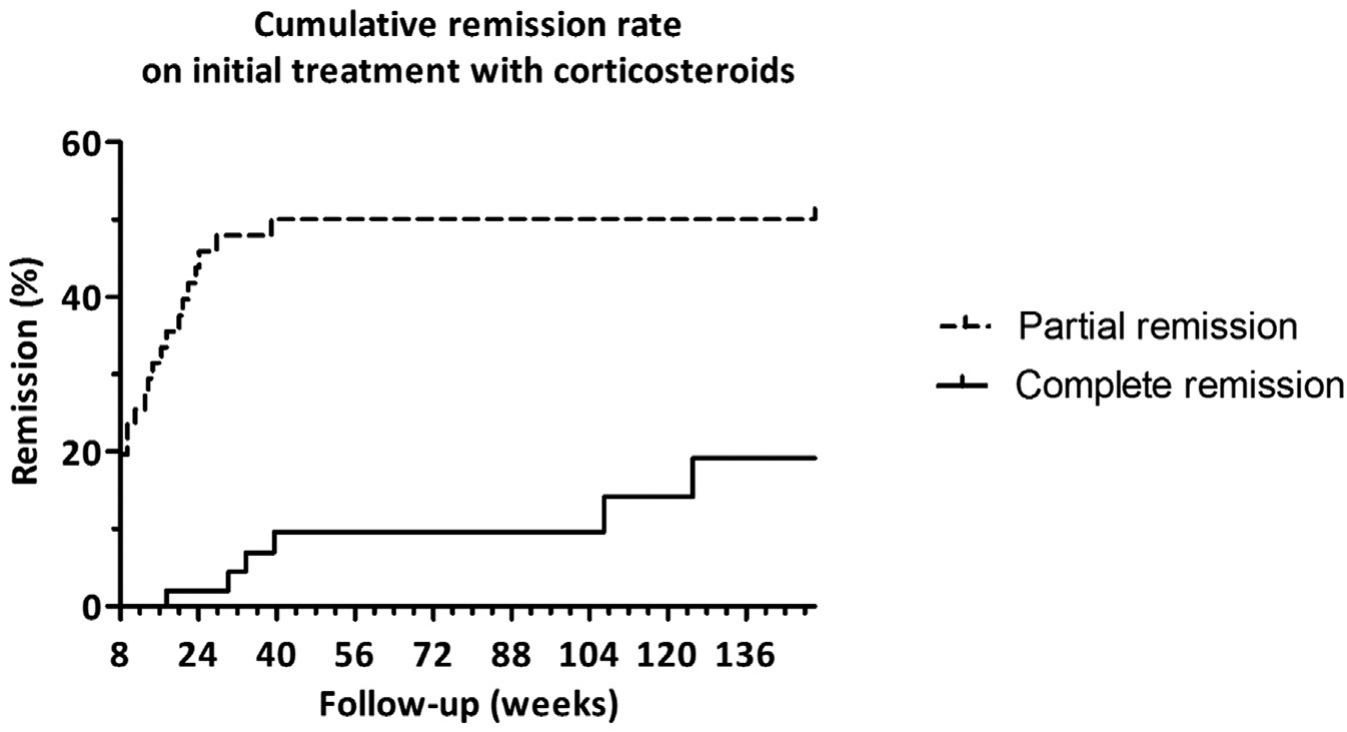
Cumulative remission rate on initial treatment with corticosteroids. Patients were censored when partial or complete remission was attained, when the patient received additional immunosuppressive therapy, or at end of follow-up. High-dose corticosteroids were continued for a median of 17 (IQR 11–21) weeks, with a total duration (including tapering) of 56 (IQR 28–83) weeks. IQR, interquartile range.

**Figure 2:**
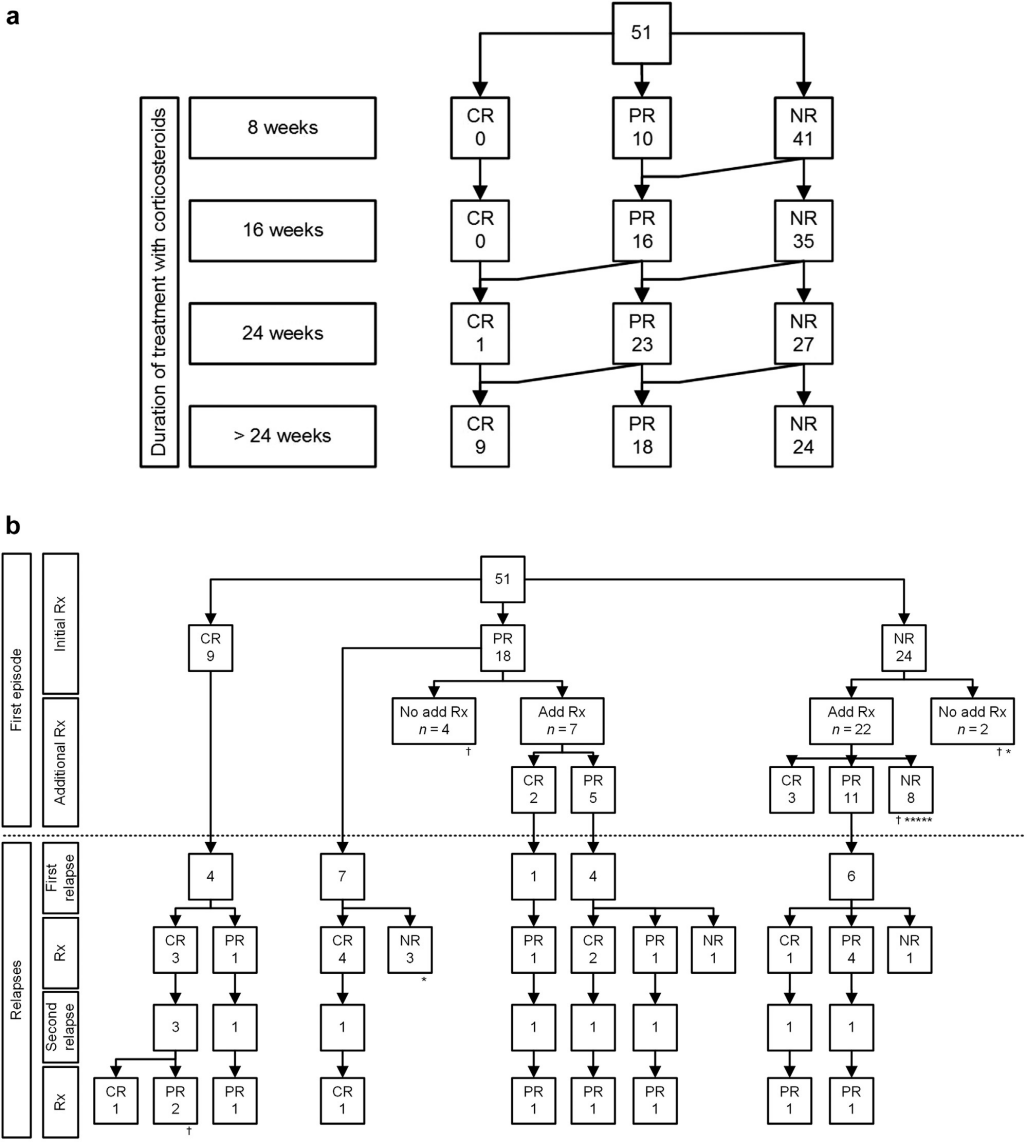
(a) Results of treatment of first episode of nephrotic syndrome with corticosteroids. (b) Results of treatment of first episode of nephrotic syndrome (upper part) and treatment of relapses (lower part). Symbols: † death; ∗ ESRD (each symbol represents 1 patient). CR, complete remission; ESRD, end-stage renal disease; NR, no remission; PR, partial remission; Rx, immunosuppressive treatment.

All patients were advised to use a sodium-restricted diet, diuretics were given to treat edema, and blood pressure was treated preferably with an angiotensin-converting enzyme inhibitor. In this study, 48 patients (94%) were using an angiotensin-converting enzyme inhibitor. Treatment with CSs was started at a median of 8.9 weeks (IQR 2.9–27.7 weeks) after kidney biopsy. Only 10 of 51 patients had developed PR at 8 weeks. High-dose CSs monotherapy was continued for a median of 17 weeks (IQR 11–21 weeks), with a total duration of CSs (including tapering) of 56 weeks (IQR 28–83 weeks). The median estimated cumulative dosage prednisolone beyond 8 weeks (until complete stop of CSs or until start of additional treatment) was 6960 mg (IQR 4935–9890 mg), and the total median cumulative dose was 11,440 mg (IQR 9415–14370 mg). The cumulative incidence of CR and PR was 0 and 16 patients at 16 weeks and 1 and 23 patients at 24 weeks of treatment, respectively (Figures 1 and 2a). In total, 9 patients (18%) attained a CR and 18 patients (35%) attained a PR with (continued) treatment with CSs and without treatment with additional IS agents (Figure 2a). Of note, in some patients, lowest levels of proteinuria were reached after tapering of steroid therapy.

There were 24 patients who did not attain a CR or PR, of whom 1 patient died after 13 weeks of treatment owing to an aortic dissection before attaining remission. A second patient reached ESRD after 6 weeks of treatment. This patient started treatment after many years of observation with NS. Renal function was already severely compromised at that time point (eGFR 23 ml/min per 1.73 m^2^). It was not possible to further classify these 2 patients as a (non)responder.

### Additional IS Treatment in Patients With Persistent Proteinuria (No Relapse)

Of 18 patients who attained a PR after treatment with CSs, 7 patients were subsequently treated with additional IS therapy, resulting in CR in 2 patients (Figure 2b). The first additional course of treatment consisted of a calcineurin inhibitor (CNI) (*n* = 3), cyclophosphamide (*n* = 2), azathioprine (*n* = 1), or pulses methylprednisolone (*n* = 1). Additional treatment was started on 17 weeks (IQR 11–28 weeks) after attaining PR and 37 weeks (IQR 23–43 weeks) after start of treatment with CSs.

There were 22 patients with persistent nephrotic-range proteinuria who received additional IS therapy. The first additional course of treatment consisted of a CNI (*n* = 13), cyclophosphamide (*n* = 8), or a new course of high-dose CSs (*n* = 1). Additional treatment was started at a median of 21 weeks (IQR 17–34 weeks) after start of CSs. There were 3 patients who attained a CR and 11 who attained a PR. Of 22 patients, 8 did not respond to additional treatment and were defined as primary nonresponders.

### Recurrence of NS

During follow-up, 22 patients had 1 (*n* = 12) or more (*n* = 10) relapses (Figure 2b). Relapse rate was higher in patients who attained PR (63%) than in those with CR (36%). Treatment of relapse was variable and included CSs and/or cyclophosphamide in most patients. Although treatment was effective in most patients, 5 patients did not respond (secondary nonresponders). None of these patients had been in CR. The outcome after treatment of relapse was remarkably variable: 6 patients who had been in CR responded, but only 4 attained CR. In contrast, 16 patients who had been in PR responded, and of these, 6 reached CR (Figure 2b).

### Long-Term Outcome

**Figure 3:**
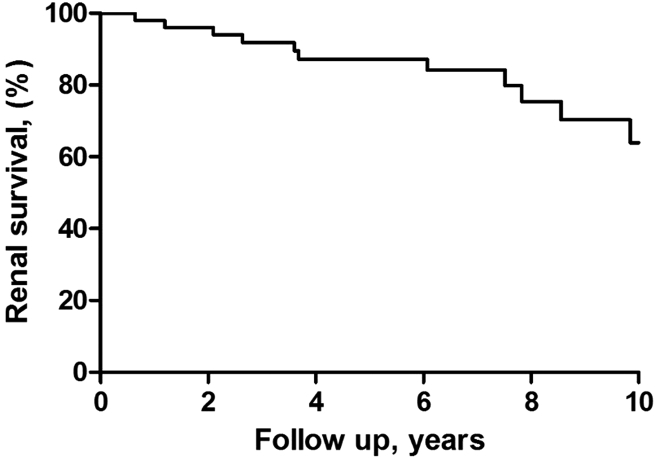
Renal survival. Renal survival at 3, 5, 7, and 10 years is 92%, 87%, 84%, and 64%, respectively.

Median follow-up was 7.1 (IQR 4.2–9.8) years. During follow-up, 4 patients died (Figure 2b). Causes of death were diverticulitis with perforation, aortic dissection, and presumed cardiovascular event (*n* = 2). There were 7 patients who have developed ESRD, 6 primary nonresponders and 1 secondary nonresponder. Renal survival was 92% at 3 years, 87% at 4 years, and 64% at 10 years (Figure 3).

During follow-up, additional 15 patients had evidence of kidney function deterioration (defined as >15 ml/min per 1.73 m^2^ decrease of eGFR and an increase of at least 1 CKD stage) but without need of replacement therapy. At the end of follow-up, in the evaluable patients (thus excluding patients who had died or developed ESRD), proteinuria level was 1.2 g (IQR 0.4–3.0 g/24 hours or g/10 mmol creatinine) (Supplemental Table S1).

In total, 4 patients (all primary nonresponders) received a kidney transplantation. In addition, 2 patients were diagnosed with having genetic FSGS and did not have recurrence of FSGS in the transplanted kidney. Of the remaining 2 patients with a kidney transplantation, 1 had a recurrence of FSGS and was treated with additional IS treatment and plasmapheresis.

### Characteristics Associated With Renal Outcome

**Figure 4:**
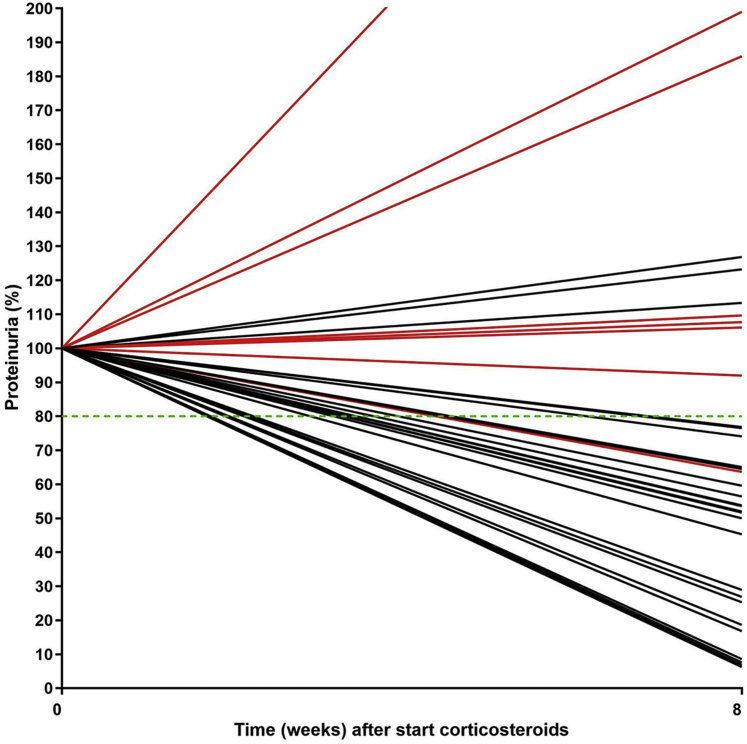
Proteinuria at start of therapy and 8 weeks. Red lines: primary nonresponders; black lines: responders; green line: cutoff of 80%. Missing data: *n* = 15 (no data at start of corticosteroids or at week 8). Patients who were not evaluable (*n* = 2) were excluded.

Paired data of proteinuria at start of therapy and after 8 weeks were evaluable in 34 patients. Patients who were not evaluable as a (non)responder (*n* = 2) were excluded. A decrease of proteinuria level >20% at 8 weeks after start of high-dose CSs predicted a response to IS therapy (to CSs/alternative IS therapy) (Figure 4). Only 1 of 24 patients with a decrease of proteinuria level of >20% seemed to be a primary nonresponder. In contrast, 10 patients had a decrease of proteinuria level of <20%. Of these patients, 7 were primary nonresponders.

**Table:** 

	Primary nonresponders (*n*)	Responders (*n*)	Total
<20% decrease proteinuria	7	3	10
>20% decrease proteinuria	1	23	24
Total	8	26	34

Progression of kidney function deterioration in patients who initially responded to IS therapy (*n* = 41) was further evaluated (Table 2). Of 41 patients, 15 patients had a decrease of eGFR > 15 ml/min per 1.73 m^2^ (and at least 1 CKD stage higher). Median age in these patients tends to be younger (45.6 [IQR 33.2–53.8] years) compared with patients with a stable kidney function (52.9 [IQR 38.4–66.6] years; *P* = 0.13). Patients with kidney function deterioration had a more preserved kidney function than those without deterioration (*P* = 0.03). There were no differences in serum albumin, proteinuria, or pathologic subtype. There was however a significant difference in the type of IS treatment, with more patients with kidney function deterioration having received CNIs (73% (*n* = 11) versus 35% (*n* = 9); *P* = 0.02). In 8 of these 11 patients, kidney function decline occurred mainly at time of usage, suggesting CNI toxicity. After cessation of the CNI, kidney function decline stabilized or improved in 3 patients. In 5 patients, kidney function further deteriorated after cessation of CNI. Of 11 patients, 3 also had kidney function deterioration, likely owing to CNI toxicity; however, follow-up was too short to see whether kidney function stabilized or improved after cessation of the CNI.

**Table 2:** Characteristics of responders with a stable kidney function compared with patients with deterioration (defined as decrease of eGFR >15 ml/min per 1.73 m^2^ and at least 1 CKD stage lower)

Characteristics	Kidney function deterioration		*P* value
	No	Yes	
Total (*n*)	26	15	
Baseline			
Age (yr)	52.9 (38.4–66.6)	45.6 (33.2–53.8)	0.13
Gender, female	9 (35)	5 (33)	1.00
BMI (kg/m^2^)	26.2 (24.9–28.7)	25.8 (24.8–29.5)	0.62
AKI	8 (31)	3 (20)	0.52
Serum creatinine (μmol/l)—no AKI	93.5 (86–106)	80.0 (63.5–90.5)	**0.03**
eGFR (ml/min per 1.73 m^2^)—no AKI	72.5 (53.3–90.0)	96.5 (86–103)	**0.03**
Serum albumin (g/l)	19.9 ± 7.0	22.3 ± 4.3	0.25
Urinary protein excretion (g/24 h or g/10 mmol)	8.3 (6.4–10.2)	8.6 (6.3–14.6)	0.39
Pathologic subtype			
Tip	12 (46)	7 (47)	1.00
Other subtypes (including NOS, cellular, perihilar, collapsing)	14 (54)	8 (53)	
Clinical course			
Duration of FU (yr)	6.9 ± 4.9	7.6 ± 2.9	0.61
Time between biopsy and start of CS therapy (wk)	6.6 (3.0–21.6)	9.1 (1.6–16.7)	0.31
Duration of high-dose CS therapy (wk)	17.3 (13.4–22.0)	19.0 (12.7–20.6)	0.78
Calcineurin inhibitor	9 (35)	11 (73)	**0.02**
Rituximab	1 (4)	4 (27)	0.05
Response during FU			
PR at 16 wks^a^	10 (38)	6 (40)	1.00
No PR at 16 wks			
Remission with CS only^a^	6 (23)	5 (33)	0.49
Remission with second-line therapy^b^	10 (38)	4 (27)	0.51
Secondary nonresponder	1 (4)	4 (27)	0.05
Relapse after attaining PR/CR	11 (42)	11 (73)	0.10
End of FU			
ESRD	0 (0)	1 (7)	0.36
Death	2	0	0.48
eGFR (ml/min per 1.73 m^2^)^c^	76.5 (49.3–88.5)	53.5 (36.0–73.5)	**0.04**
Serum albumin (g/l)^c^	34.3 ± 7.2	34.0 ± 8.5	0.90
Urinary protein excretion (g/24 h or g/10 mmol)^c^	0.7 (0.3–2.0)	1.2 (0.2–2.9)	0.17
<0.3	6 (25)	4 (29)	
0.3–3.5	17 (71)	7 (50)	
>3.5	1 (4)	3 (21)	

AKI, acute kidney injury; BMI, body mass index; CKD, chronic kidney disease; CR, complete remission; CS, corticosteroid; eGFR, estimated glomerular filtration rate; ESRD, end-stage renal disease; FU, follow-up; IQR, interquartile range; NOS, not otherwise specified; PR, partial remission.

Values are presented as *n* (%), mean ± SD, or median (IQR). Bold values indicate statistically significant (*P* < 0.05).

a Including 2 patients who can be classified as a secondary nonresponder.

b Including 1 patient who can be subclassified as a secondary nonresponder.

c In patients surviving without ESRD. Primary nonresponders (*n* = 8) and patients who were not evaluable (*n* = 2) were excluded.

Next, we analyzed the primary nonresponders. After exclusion of the patients with a genetic cause, the most remarkable baseline characteristic was a relatively young age (median age 29.3 years [IQR 19.3–34.3 years]). Other baseline characteristics of the primary nonresponders included a mean body mass index of 25.9 kg/m^2^ (range 21.2–34.8); an eGFR (no acute kidney injury) of 87.8 ml/min per 1.73 m^2^ (range 35–129); proteinuria 10.1 g/d (range 4.9–11.8), and a mean serum albumin 19.1/l (range 14–30).

We also analyzed remission rate according to histologic subtype. There were no significant differences in total or CR rate (Supplemental Figure S1A and B). Of note, this analysis has low power owing to low number of patients.

### Genetic Analysis

Genetic analysis was performed in 25 of 51 patients (49%). There were 6 patients who were tested in daily clinical practice and 19 patients who were tested in the context of research (Supplemental Table S2). In addition, 63% of the patients who did not attain a PR at 16 weeks of treatment were genetically tested with a bias toward poor responders in whom more frequently genetic testing was performed. In 7 of 8 (88%) primary nonresponders, genetic testing was performed and 2 patients were identified with a genetic cause: 1 patient was diagnosed with having mitochondrial encephalomyopathy, lactic acidosis, and stroke-like episodes and another patient had 2 pathogenic variants in *NPHS2* (compound heterozygous). No other genetic causes were identified in the remaining tested patients.

## Discussion

Treatment of patients with pFSGS is heavily debated. The debate is not only fostered by the lack of well-powered randomized clinical trials but largely explained by the lack of a diagnostic biomarker. FSGS is not a disease entity but rather a description of a histologic pattern of glomerular injury. A total of 4 subgroups of patients with FSGS can be defined as follows:^2^

1. Patients with presumed pFSGS. pFSGS is defined as a clinical-pathologic entity, characterized by NS, complete foot process effacement, variable responsiveness to IS therapy, and recurrence after kidney transplantation. pFSGS is caused by a permeability factor; however, the identity of this factor is as yet unknown. The absence of a biomarker does not allow to make a diagnosis of primary permeability factor-associated FSGS in patients who present with NS. Still, many would agree that patients with pFSGS can be reliably defined and diagnosed by clinical criteria.
2. Patients with genetic FSGS. In recent years, there has been an increasing knowledge and discovery of disease-causing genes. Still, there will be patients with FSGS owing to unidentified pathogenic variants (e.g., intronic, mitochondrial) or in genes either not yet discovered or known at the time of the testing. A positive family history for kidney disease could be a clue. Some of the patients with genetic FSGS clinically mimic pFSGS.
3. Patients with secondary FSGS owing to a maladaptive response or underlying kidney disease. In these patients, there is an absolute or a relative loss of podocytes, attributable to readily identifiable causes, such as nephron loss, obesity, hypertension, and diabetes mellitus.
4. Patients with FSGS of undetermined cause. These patients present with FSGS on histology, with incomplete foot process effacement, clinically characterized by (non)nephrotic-range proteinuria but without NS. These patients mimic patients with maladaptive or genetic FSGS, but the cause remains unknown.

Our study indicates that clinical criteria allow to identify patients with presumed pFSGS with reasonable but not absolute accuracy. By including only patients with evident NS, in a White population, and excluding patients with a positive family history or documented nephron loss, we selected a group of patients of whom most (cumulative incidence rate 80%) responded to IS therapy. Of note, no biopsy result revealed perihilar FSGS, and 93% of evaluated kidney biopsies had complete foot process effacement supporting the appropriateness of our selection criteria. Although our study has many limitations (see subsequent texts), inherent to a retrospective study that evaluates routine clinical care, we can draw some conclusions, as follows:

### Absence of CR at 8 and 16 Weeks of High-Dose CS Therapy Does Not Define Steroid Resistance

We excluded patients with CR within 8 weeks after start of therapy, to avoid the debate that patients with FSGS may mimic minimal change disease (especially the FSGS-tip lesion). Still, only few patients (2 of 53) developed CR within 8 weeks after start of therapy. Thus, 51 patients had persistent proteinuria at 8 weeks. Of note, even after 16 weeks, there was no additional patient with CR. Still, with prolonged follow-up, a CR developed in almost 40% of patients and CR was often only notable after second-line treatment with IS therapy or after treatment of relapse. Clearly, even the lack of CR after 16 weeks of therapy does not properly define steroid resistance. We argue that also PR at 16 weeks is not a good biomarker of treatment response. In our cohort, only 16 of 51 patients (31%) had developed PR at 16 weeks, whereas overall 80% of patients developed PR at some time during follow-up.

### It Takes Time and Long-Term IS Therapy to Develop Remission

Our patients initially received CSs, with high-dose steroids administered for 17 weeks, and total treatment duration extending beyond 52 weeks. Many patients received second-line IS therapy and only developed CR or PR thereafter. We cannot exclude that the patients who developed remission after second-line therapy might have responded to longer duration of CSs or to high-dose i.v. methylprednisolone. Still, the data indicate that many patients will need prolonged therapy and that we must consider a more aggressive approach in some patients. This is also illustrated by the observation of patients who developed CR only after being treated with renewed therapy for a relapse after PR. Our study extends earlier observations that remission rate in patients with FSGS increases with longer duration of therapy. From their literature review, Shabaka *et al.*^1^ concluded that treatment with short courses of high-dose CSs (<2 months) resulted in remission rates of only 20% to 30%. Data of previously published studies of steroid therapy in adult patients with FSGS are summarized in Supplemental Table S3. Comparison between these studies is difficult because many included patients without NS.^5^, ^6^, ^7^^,^^15^^,^^24^, ^25^, ^26^, ^27^, ^28^ Overall remission rates in these studies ranged from 40% to 63%, which is in agreement with our remission rate of 53% after initial (prolonged) steroid therapy. Although average treatment duration is seemingly short, it is important to realize that in most studies, as in our cohort, treatment duration was adapted to the clinical response, with treatment being shorter in patients who attain rapid remission. The study of Ponticelli *et al.*^4^ mirrors our findings. These authors evaluated outcome in 80 patients with FSGS. All patients were White, patients with a documented cause of secondary FSGS were excluded, and almost all patients had NS, as reflected by the mean serum albumin level of 22.4 ± 8.7 g/l. Patients were treated with glucocorticoid monotherapy (*n* = 53) or with other IS drugs combined with steroids (*n* = 27). High-dose steroid therapy was given until CR or at least 8 weeks, with gradual tapering thereafter for a total treatment duration of at least 6 months. Patients receiving other IS agents were often treated for >12 months. Patients who did not respond at 26 weeks were offered retreatment. Overall, 52% of patients developed CR or PR after initial therapy. Moreover, of 38 nonresponders, 26 were retreated, and of these, 15 developed remission. The cumulative remission rate in the treated patients was 84%, and after 110 months, 69% of the patients were alive with maintained renal function (without doubling of serum creatinine level). Thus, this study already revealed that clinical criteria enable the selection of patients who are responsive to immunosuppression, although prolonged therapy is needed to induce remission. Of note, recent studies that questioned a high response rate to IS therapy in FSGS included many patients without NS.^15^^,^^29^

### Histologic FSGS Criteria Are of Limited Value in Presumed pFSGS

The Columbia classification is often used to classify FSGS. In our study, 49% of the patients had FSGS subtype not otherwise specified, 39% a tip-lesion, whereas the other subtypes were rare. Although our study is underpowered, the data indicate that the type of FSGS lesion had no major impact on steroid responsiveness nor long-term outcome. In another study, Sethi *et al.*^30^ revealed that FSGS subtype not otherwise specified did not differentiate between pFSGS and secondary FSGS. Taken together, the data question the clinical relevance of the Columbia classification.

### A Clinical Diagnosis of pFSGS Does Not Completely Exclude a Genetic Cause

Genetic analysis was only done in a limited number of patients, and in some cases, genetic analysis was limited to single-gene mutation analysis (Supplemental Table S2). A genetic cause was identified in 2 patients of 25 tested patients (8%). If we restrict the analysis to the overall nonresponders, detection rate is 25%. Because there was a bias in our study toward more genetic testing in patients with poorer response, we expect that the likelihood of detecting a causative mutation in adult patients with NS in the absence of known family history is <8%. This percentage is in agreement with Miao *et al.*^31^ These authors investigated the characteristics of 49 patients with FSGS with the purpose to find characteristics that could increase the likelihood of finding a causative genetic variant. In total, 13 patients were defined as having presumed pFSGS (these patients had NS and complete FPE in EM of the renal biopsy). Only 1 patient had a causative genetic variant.^31^ If we compare the results of Miao *et al.*^31^ and our study with those of other studies, the percentages are relatively low, presumably owing to inclusion of patients without nephrosis in other studies. Gribouval *et al.*^32^ investigated 135 patients with sporadic steroid-resistant NS and/or FSGS with a mean proteinuria of 7.1 g/d (range 1.8–18.0) and mean serum albuminemia of 22.3 g/l (range 8.0–41.0). Mean age at diagnosis of proteinuria was 30.3 (18.1–84.0) years. A (likely) pathogenic variant was identified in 16 patients (12%). Of these 16 patients, only 3 patients had a serum albumin level <30 g/l, and in 9 patients, serum albumin level was not reported. Gast *et al.*^33^ investigated 69 cases of adult-onset FSGS (≥18 years) and 10 cases with onset below the age of 18 years. In total, 67 patients had no positive family history and only 35 patients had NS. In 10% of the sporadic cases, a genetic cause was identified, but it is unknown whether these patients presented with an overt NS.

### CNI in FSGS: A Double-Edged Sword?

Guidelines advocate the use of CNI in patients with steroid-resistant NS. CNI can also be used as steroid-sparing agents in patients with frequently relapsing NS or steroid-dependent NS.^8^ Furthermore, in our clinical practice, CNI has been used. Our study does not allow conclusions on efficacy in comparison with other drugs. Still, we noted that the use of CNI was associated with decreased eGFR. We caution against overzealous use of CNI and suggest that CNI withdrawal must be considered in patients with deteriorating eGFR.

### Better Biomarkers Are Needed to Predict (Non)Response

We investigated change in proteinuria within 8 weeks after start of therapy. A cutoff of a decrease of proteinuria >20% predicted response to IS therapy. Therefore, we suggest that a decrease of >20% warrants continuation of IS therapy. De Vriese *et al.*^34^ recently discussed the relevance of genetic testing in patients with steroid-resistant FSGS. Our data suggest that a decrease of <20% could serve as a moment to consider genetic testing, which also could be a cost-effective approach. Of note, 3 patients who had initially no decrease of >20% responded also to IS therapy. Therefore, more biomarkers (besides genetic testing) are needed to predict response to therapy.

Progression of kidney function deterioration in patients who responded to IS therapy (*n* = 41) occurred in 15 patients (Table 2). Remarkably, these patients had a better-preserved kidney function at baseline, probably because these patients were younger. The younger age in combination with kidney function deterioration could suggest a genetic predisposition in this group. Another explanation might be that CNIs are more easily used in patients with preserved eGFR.

In our study, up to 20% of patients (at least 8 patients, possibly 10) did not respond to therapy, consisting of CSs and second-line IS therapy. Only 2 patients had proven genetic FSGS, which in retrospect explained steroid resistance. After exclusion of the patients with a genetic cause, the most remarkable baseline characteristic was a relatively young age (median age 29.3 years [IQR 19.3–34.3 years]). The younger age suggests a genetic unidentified cause, because most patients with genetic FSGS present at younger age.^32^^,^^33^ Nevertheless, we cannot exclude that longer or more aggressive therapy might have been more effective, but we consider it unlikely. Alternatively, these patients may have secondary FSGS owing to other unknown causes (virus, drugs), combined with maladaptive response because of low nephron number. Early recognition of these patients may reduce the exposure to toxic drugs.

Obviously, our study has limitations. The data were extracted retrospectively, treatment was done by discretion of the treating physician, genetic analysis was done in a subgroup only, and EM was unavailable in 43% of the patients. Because patients were recruited from different centers in a long-term period, different assays were used to measure serum albumin. Moreover, in the absence of a valid biomarker, the diagnosis of pFSGS remains debatable. The outcome of a decrease of proteinuria level >20% should be validated in a different cohort.

Long-term use of high-dose CSs is associated with side effects. Because our study was retrospective, there was no protocolized reporting of adverse events. We expect that many patients will have suffered from the many side effects associated with the use of steroids, such as obesity, Cushingoid face, skin bruising, and mood disturbances. In addition, because many patients used steroids for a long period of time, we suspect that few patients experienced side effects that necessitated withdrawal of steroids. In the study of Ponticelli *et al.*,^4^ severe side effects, including infections and diabetes, occurred in approximately 10% of patients. In a separate analysis of the effects of steroid therapy in patients with minimal change disease, observed side effects included CS-induced diabetes mellitus in 7% of patients, osteoporotic fracture in 2%, and CS-induced psychosis in 2%.^35^ We suggest that additional (steroid sparing) IS therapy should be considered whenever steroid side effects become apparent.

In conclusion, outcome is not necessarily dismal in patients with NS owing to presumed pFSGS. Outcome is worse in patients with an ongoing NS despite treatment with IS agents. Most of the patients with ongoing NS had negative result for a genetic cause. The heterogeneous retrospective cohort studies in literature underline the need for better clinical definitions to define pFSGS. Our study reveals that an overt NS and absence of secondary causes include many patients with pFSGS, but this definition is not sufficient enough. We argue that genetic testing should be performed in patients with an overt NS (and absence of positive family history) when proteinuria decreased <20% within the first 8 weeks of IS treatment. Moreover, future studies should focus on the discovery of clinical characteristics and/or biomarkers that identify patients with pFSGS who will and will not benefit from IS therapy. Recently, Agrawal *et al.*^36^ proposed a panel of 3 cytokines (interleukin-7, interleukin-9, and MCP-1) as biomarker to predict steroid resistance before treatment with CSs; however, further studies should validate these findings.

## Disclosure

IMR was supported by an AGIKO-grant of ZonMw-NWO (grant 92003587). The research leading to the genetic results in this manuscript has received funding from the European Community’s Seventh Framework Program under grant agreement number 2012-305608 “European Consortium for High-Throughput Research in Rare Kidney Diseases (EURenOmics).” Several authors of this publication are members of the European Reference Network for Rare Kidney Diseases (ERKNet)–project identification number 739532. All the other authors declared no competing interests.

## Supplementary Material

Supplementary File (PDF)

**Figure S1.** (A) Cumulative rate of complete remissions on initial treatment with corticosteroids in patients according to type of histologic lesion. (B) Cumulative rate of remissions on initial treatment with corticosteroids in patients according to type of histologic lesion.

**Table S1.** CKD stage at end of follow-up.

**Table S2.** Overview of genetic testing.

**Table S3.** Response to treatment with corticosteroids in patients with a nephrotic syndrome owing to FSGS: overview of literature.
