# Supplementary material for: Later Response to Corticosteroids in Adults With Primary Focal Segmental Glomerular Sclerosis Is Associated With Favorable Outcomes
Source: Kidney Int Rep. 2021 Oct 29;7(1):87–98. doi: 10.1016/j.ekir.2021.10.016 (PMC8720814; doi:10.1016/j.ekir.2021.10.016)
Supplement: Supplementary File (PDF) [file mmc1.pdf]

Supplementary Material

Supplemental Figure s1a Cumulative rate of complete remissions on initial treatment with corticosteroids in patients according type of histologic lesion.

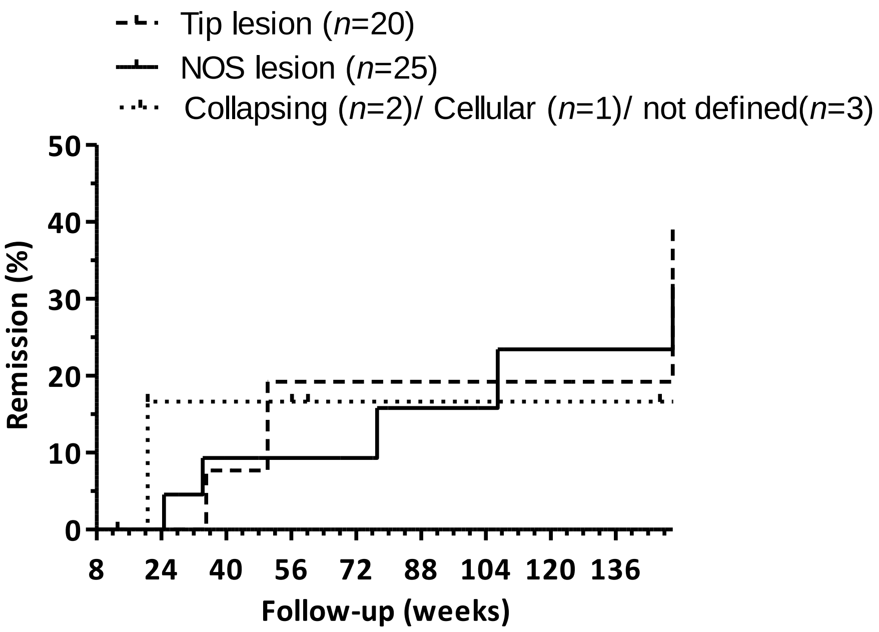

Supplemental Figure s1b Cumulative rate of remissions on initial treatment with corticosteroids in patients according type of histologic lesion.

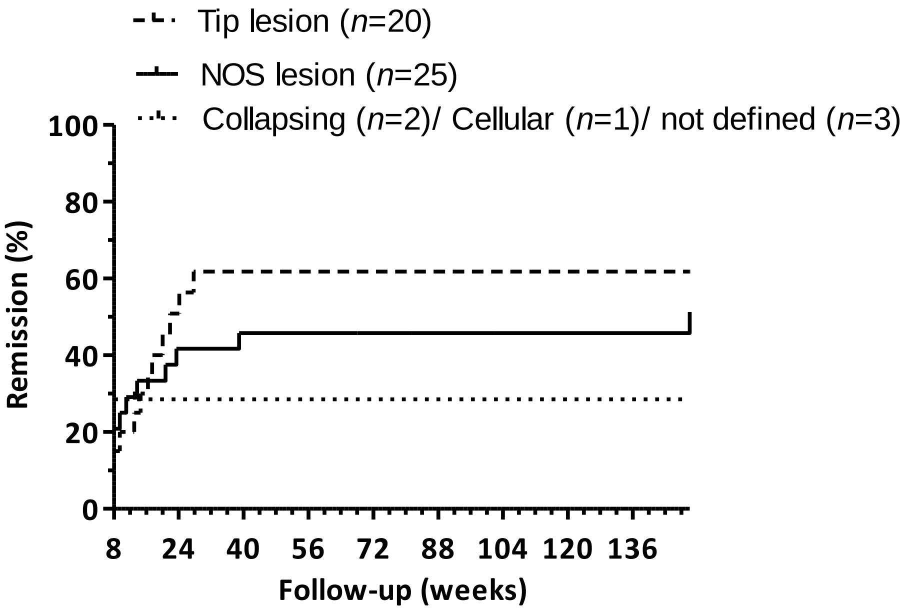

Supplemental Table s1. CKD stage at end of follow-up.

	A1	A2	A3		
	(<30mg)	(30-300mg)	(0.3-1gr)	(1-3.5gr)	(>3.5gr)
G1	1	2	1	3	1
G2	3	3	3*	7	2*
G3a	0	1	2	3	1*
G3b	0	0	4*	1	1
G4	0	1	0	2	2
G5	---------------- 7 ----------------				

Stage A3 was subdivided into three categories, encompassing respectively 0.3-1gr/ 10 mmol creatinine /per 24 hours, 1-3.5gr/ 10 mmol creatinine or per 24 hours and >3.5gr/ 10 mmol creatinine or per 24 hours. The last available values were used for the patients who died (indicated with an Asterix (one asterix indicates one patient).

Supplemental Table s2. Overview of genetic testing.

**Clinical features**	**Genetically tested patients (%)**	**Genetic testing (n)**		**Type of genetic test**	**Results**
Response during FU (total group)		Routine	Research		
PR at 16 weeks (n=16)[a]	19%	1	2	Targeted seq (n=2)	Negative
				Sanger seq *NPHS2* gene (n=1)	Negative
No PR at 16 weeks					
Remission with CS only (n=11)[a]	27%	2	1	Targeted seq (n=1)	Negative
				WES (n=2)	Negative
Remission with 2nd line therapy (n=14)[b]	79%	1	10	Targeted seq (n=10)	Negative
				WES (n=1)	Negative
Primary non-responder (n=8)	88%	2	5	Targeted seq (n=4)	Negative
				Sanger seq *NPHS2* gene (n=1)	Negative
				Mitochondrial mutations (n=1)	MELAS[c]
				WES (n=1)	*NPHS2* p.(Arg138Gln) htz and p.(Val338Met) htz
Not evaluable (n=2)	50%		1	Targeted seq (n=1)	Negative
Secondary non-responder (n=5)	80%	2	2	Targeted seq (n=2)	Negative
				WES (n=2)	Negative

[a] Including two patients who can be classified as a secondary non-responder; [b] including one patient who can be subclassified as a secondary non-responder; [c] this patient was published previously[1] Abbreviations: PR: partial remission; CS: corticosteroids; seq: sequencing; WES: whole exome sequencing; htz: heterozygous; MELAS: Mitochondrial Encephalomyopathy, Lactic Acidosis, and Stroke-like episodes (m.3243A>G).

Supplemental Table s3. Response to treatment with corticosteroids in patients with a nephrotic syndrome due to FSGS: overview of literature.

If information available, we reported only information about patients with a nephrotic syndrome who were treated with cortico steroids. If no detailed information was available, we reported information based on total cohort, including non-nephrotic patients (indicated with *). Abbreviations: P: proteinuria; S c: serum creatinine; NS: nephrotic syndrome; CS: corticosteroids; CR: complete remission; PR: partial remissio n.

Article	Study type	Patients with FSGS and NS *n* (total cohort (*n*))	Patients with NS initially treated with CS, *n* (%)	Follow-up, mean ± SD or median (range) years	Baseline characteristics			
					Age, years (mean)	Serum creatinine, mg/dL (mean ± SD) or median (range))	Serum albumin, g/dL (mean ± SD)	Proteinuria, g/24 hours or g/10mmol creatinine, (mean ± SD or median (range))
Ponticelli et al. 1999[2]	Retrospective cohort	80 (80)	53/80 (66)	7.2 (1-28.5)*	40.8 ± 19.1	1.09 ± 0.37	2.4 ± 0.9	6.96 ± 4.36
Alexopoulos et al. 2000[3]	Retrospective cohort	17 (33)	11/17 (65)	4.6 (0.7-11.8)*	41 (14-77)*	1.9 ± 1.5*	N/A	3.5 ± 3.3*
Rydel et al. 1995[4]	Retrospective cohort	60 (81)	30/60 (50)	5.2 ± 5.4	38.1 ± 16	2.3 ± 1.8	N/A	10.4 ± 8.3
Pei et al. 1987[5]	Retrospective cohort	30 (55)	13/30 (43)	N/A	35	N/A	N/A	N/A
Goumenos et al. 2006[6]	Retrospective cohort	33 (51)	9/33 (of which 8 with NS)	5*	37 ± 16*	1.22 ± 0.4*	2.8 ± 0.8*	5.8 ± 6.6*
Bolton et al. 1977[7]	Retrospective cohort	10 (82)	10/10 (100)	2.8 ± 0.7	28.9 ± 4.6	1.53 ± 0.23	2.6 ± 0.3	6.6 ± 1.0
Stirling et al. 2005[8]	Retrospective cohort	136 (136)	76/136 (56)	N/A	40.9 ± 17.0	1.29 (0.96-1.92)	2.2 ± 6.7	10 (6-15)
Fernandez-Juarez et al. 2016[9]	Retrospective cohort	48 (119)	40/48 (83)	3.7 (IQR 1.8-4.7)*	44.3 ± 19.0*	1.37 ± 0.7*	2.8 ± 1.0*	7.0 ± 5.8*
Cattran et al. 1998[10]	Retrospective cohort	17 (55)	17/17 (100)	10.5	35*	N/A	N/A	N/A
Agarwal et al. 1993 [11](abstract only)	Retrospective cohort	38 (65)	38/38 (100)	2.7	N/A	N/A	N/A	7.5 ± 4.3
Chitalia et al. 1999[12]	Retrospective cohort	N/A (111)	28/111 (25)	10.8 ± 8.5	35.6 ± 14.9*	1.47 ± 0.79	3.9 ± 0.8*	4.9 ± 4.0*
Current study	Retrospective cohort	51 (51)	51/51 (100)	7.1 (4.2-9.8)	45.9 ± 17.2	1.06 (0.87-1.59)	2.1 ± 0.6	8.7 (6.3-12.7)

Table S3 continuation.

Article	Definition CR	Definition PR	PR reached on CS, n (%)	CR reached on CS, n (%)	Duration of CS therapy, median months	Relapse after CS induced remission n (%)	PR or CR at end of FU n (%)	Occurrence of ESRD, n (%)	Remarks
Ponticelli et al.1999	P: <0.2g/d S c: <1.5mg/dL	P: 0.21-2g/d S c: <1.5mg/dL	10/53 (19)	21/53 (40)	3.7 (1.8 months high dose)	N/A	60 (75)*	17 (22)* doubling of serum creatinine	-
Alexopoulos et al. 2000	P: <0.25/d S c: <1.5mg/dL	P: 0.26-3g/d S c: stable and <1.5mg/dL	4/11 (36)	3/11 (27)	9 (=mean) (at least 1 month high dose)	0/11 (0)	9/11 (82)	0/11 (0)	-
Rydel et al. 1995	P: <0.25/d	P: 0.26-2.5 g/d	5/30 (17)	10/30 (33)	5.5 ± 4 (at least 1 month high dose in 26/30 patients)	10/15 (67)	14 /30 (47)	6/30 (20)	- Three patients received additional cytotoxic therapy during initial treatment<br>- 63% of patients was black.<br>- all patients who progressed to ESRD were non-responders
Pei et al. 1987	P: <0.25/d with stable kidney function for 1 year	N/A	N/A	5/13 (38)	N/A	N/A	N/A	N/A	- 58% of nephrotic patients were not treated.
Goumenos et al. 2006	P: <0.3g/d	P: 0.3-3g/d	5/8 (63)	N/A	16 (=mean) (at least 4 months high dose)	1/5 (20)	N/A	N/A	- Unclear whether remission rate of 5/8 concerned a partial and/or complete remission.<br>- Of the 51 patients within this cohort, 25 were treated. Baseline characteristics that are shown concern data of the treated patients.
Bolton et al. 1977	P: <0.2g/d	P: 0.2-3gr/d	4/10 (40)	0/10 (0)	21.4 ± 7.1	N/A	3/10 (30)	1/10 (10)	- mean dosage every other day was 72.2± 8.6mg prednisone.
Stirling et al 2005	P: <02g/d	P: 0.2-2g/d	N/A	N/A	14	N/A	50/76 (66)	N/A	- 45 patients had more than one agent in addition to prednisone over the treatment period.<br>- no patient received >3 months a dosage above 40mg/d
Fernandez-Juarez et al. 2016	P: <0.3g/d S c: normal	P: 0.3-3.5g/d and >50% reduction	16/40 (40)	N/A	12.8 ± 9.0 (high dose 3.0 ± 4.5)	16/40 (40)	N/A	N/A	- unclear whether remission rate of 16/40 concerned a partial and/or complete remission.

	S alb: >3.5g/dL	S c: stable							
Cattran et al. 1998	P: <0.2g/d S c: stable	N/A	N/A	8/17 (47)	5 (2-50)	3/8 (38)	N/A	5/17 (29)	-
Agarwal et al. 1993 (abstract only)	N/A	N/A	10/38 (26)	12/38 (32)	N/A	N/A	N/A	N/A	-
Chitalia et al. 1999	P: <0.3g/d	N/A	N/A	6/28 (21)	Max 3 months (high dose 1.0 mg/kg for 6 to 8 weeks)	N/A	30 (29)*	49 (44)*	- Based on baseline data, only few patients with nephrotic syndrome were included.
Current study	P: ≤0.3g/d with increasing s albumin	P: 0.3-3.5g/d and >50% reduction S c: stable	18 (35)	9 (18)	14 (4.2 high dose)	22 (46)	35 (69)	6 (12)	

References

1. Lowik MM, Hol FA, Steenbergen EJ*, et al.* Mitochondrial tRNALeu(UUR) mutation in a patient with steroid-resistant nephrotic syndrome and focal segmental glomerulosclerosis. *Nephrol Dial Transplant* 2005; **20:** 336-341.

2. Ponticelli C, Villa M, Banfi G*, et al.* Can prolonged treatment improve the prognosis in adults with focal segmental glomerulosclerosis? *American journal of kidney diseases : the official journal of the National Kidney Foundation* 1999; **34:** 618-625.

3. Alexopoulos E, Stangou M, Papagianni A*, et al.* Factors influencing the course and the response to treatment in primary focal segmental glomerulosclerosis. *Nephrol Dial Transplant* 2000; **15:** 1348-1356.

4. Rydel JJ, Korbet SM, Borok RZ*, et al.* Focal segmental glomerular sclerosis in adults: presentation, course, and response to treatment. *American journal of kidney diseases : the official journal of the National Kidney Foundation* 1995; **25:** 534-542.

5. Pei Y, Cattran D, Delmore T*, et al.* Evidence suggesting under-treatment in adults with idiopathic focal segmental glomerulosclerosis. Regional Glomerulonephritis Registry Study. *Am J Med* 1987; **82:** 938-944.

6. Goumenos DS, Tsagalis G, El Nahas AM*, et al.* Immunosuppressive treatment of idiopathic focal segmental glomerulosclerosis: a five-year follow-up study. *Nephron Clin Pract* 2006; **104:** c75-82.

7. Bolton WK, Atuk NO, Sturgill BC*, et al.* Therapy of the idiopathic nephrotic syndrome with alternate day steroids. *Am J Med* 1977; **62:** 60-70.

8. Stirling CM, Mathieson P, Boulton-Jones JM*, et al.* Treatment and outcome of adult patients with primary focal segmental glomerulosclerosis in five UK renal units. *QJM : monthly journal of the Association of Physicians* 2005; **98:** 443-449.

9. Fernandez-Juarez G, Villacorta J, Ruiz-Roso G*, et al.* Therapeutic variability in adult minimal change disease and focal segmental glomerulosclerosis. *Clin Kidney J* 2016; **9:** 381-386.

10. Cattran DC, Rao P. Long-term outcome in children and adults with classic focal segmental glomerulosclerosis. *American journal of kidney diseases : the official journal of the National Kidney Foundation* 1998; **32:** 72-79.

11. Agarwal SK, Dash SC, Tiwari SC*, et al.* Idiopathic adult focal segmental glomerulosclerosis: a clinicopathological study and response to steroid. *Nephron* 1993; **63:** 168-171.

12. Chitalia VC, Wells JE, Robson RA*, et al.* Predicting renal survival in primary focal glomerulosclerosis from the time of presentation. *Kidney international* 1999; **56:** 2236-2242.
